# Occupational Therapy Acute Care Self-Efficacy Scale (OTACSES): Development, Test–Retest Reliability, and Precision

**DOI:** 10.1155/oti/8848379

**Published:** 2025-07-10

**Authors:** Corey McGee, Hannah Oldenburg

**Affiliations:** University of Minnesota, Minneapolus, Minnesota, USA

**Keywords:** acute care, measurement, occupational therapy, precision, reliability, self-efficacy

## Abstract

**Introduction:** Delivering occupational therapy services in the acute care setting requires certain skills and abilities to ensure safety and effectiveness among a variety of medically complex populations. Presently, no standardized tool exists to assess OT students' self-efficacy in this context. In this study, we developed the O*ccupational Therapy Acute Care Self-Efficacy Scale* (*OTACSES*) to assess OT student self-efficacy in acute care contexts and tested its test–retest reliability and precision in first-year OT students.

**Materials and Methods:** Researchers developed the scale items based on literature, expert interviews, and a student population. The OTACSES was then administered to 47 OT students to assess the internal consistency of “abilities” and “knowledge” subscales. Following the process of item reduction, the scale was readministered a week later after a no “intervention” control period. Student test and retest data were analyzed to assess reliability and precision.

**Results:** The finalized knowledge and abilities subscales demonstrated excellent internal consistency. Further, the total and subscale scores demonstrated excellent test–retest reliability and acceptable precision. Finally, changes of five, three, and seven points in the abilities, knowledge, and total scores, respectively, can be assumed to be “meaningful.”

**Discussion:** The OTACSES is a reliable and precise tool for measuring students' self-efficacy in the acute care setting. We present preliminary data on clinically meaningful change. Further research focusing on the tool as an outcome measure of the efficacy of acute care-relevant OT education and its appropriateness for use among practitioners is needed.

## 1. Introduction

Occupational therapy services in acute care are focused on improving a patient's functional status due to a rapid decline in their medical status or from an expected medical event [1]. According to the 2023 American Occupational Therapy Association (AOTA) workforce survey, 22.1% of occupational therapy practitioners are employed and delivering occupational therapy services in a hospital setting [2]. In the acute care setting, occupational therapy practitioners are knowledgeable in the evaluation, treatment, and outcome process that is focused on engagement in occupation as a vehicle for health and wellness [3]. Practitioners assist their patients in returning to occupations that are expected or required to be completed at a given assist level that matches the discharge location and assistance level. Skilled therapeutic interventions such as self-care retraining, adaptation or compensation teaching with the use of new medical conditions impacting function, and caregiver education and training are primary interventions facilitated by practitioners in acute care [4]. When delivering occupational therapy services, considerations for a patient's medical and procedure status are influential to the practitioner's decision making to initiate, sustain, or discontinue delivery of occupational therapy services throughout a patient's hospital duration [5]. Occupational therapy practitioners working in acute care are expected to identify, analyze, and manage medical acuity considerations for each assigned patient. Vital monitoring and laboratory value analysis are required to be completed before and during patient interactions to ensure feasibility and safety of delivering occupational therapy services to a variety of medically complex conditions and precautions [5].

### 1.1. Training

Occupational therapy practitioners' knowledge, skills, and behaviors to manage medical conditions, precautions, and considerations is essential for successful and safe participation in occupational therapy sessions in acute care [1, 5]. For occupational therapy students, fieldwork experiences in acute care provide opportunities for students to gain knowledge and skills related to acute care cultural expectations and routines, such as using life-sustaining equipment during therapy session, managing medical acuity and precaution procedures, and intraprofessional communication methods within a healthcare team [4, 5]. The knowledge, skills, and behaviors associated with caring for medically unstable populations is different than other postacute rehabilitation settings where occupational therapy delivers services.

Occupational therapy practitioners are expected to be knowledgeable and independent in understanding a variety of medical conditions, following surgical or procedural precautions, using medical and environmental equipment as well as monitoring lab and vitals while engaging patients in a variety of occupations bedside [5]. Understanding occupational therapy students' perceptions of their abilities in managing acute care skills, knowledge, or behaviors could further support education or training in occupational therapy programs as well as on-boarding of entry-level practitioners to that setting. Using a valid and reliable tool to measure practitioner self-efficacy specific to acute care practice–related abilities and knowledge may assist in identifying gaps in education or training within occupational therapy education.

Bandura [6] postulates that persons develop self-efficacy through four major sources of information: mastery experiences, vicarious experiences, verbal persuasion, and emotional arousal. Clinical simulation provides learning opportunities through utilizing these building blocks of self-efficacy. For example, experiential learning enables students to practice and obtain mastery of classroom knowledge [7]. Furthermore, Bandura argues that self-efficacy influences behavior through determining what goals individuals set for themselves and how much effort and persistence persons choose to employ in the pursuit of their goals [6].

Perceived self-efficacy as a predictor of professional practice behaviors is generally measured via questionnaires utilizing rating scales [8]. In a study investigating the impact of transfer training on self-efficacy in OT students, assessment subscales measured self-efficacy in the domains of knowledge, skills, and safety [9]. Self-efficacy ratings were gathered three times at approximately 2-week intervals after a classroom simulation experience, a first acute care simulated experience, and a second acute care simulated experience. Though self-efficacy ratings increased over time for all groups, the participant dominant group reported an increase in knowledge self-efficacy compared with the observation dominant and participation only groups. These results support Bandura's construct that vicarious learning through observation, combined with active learning through participation, produces measurable change in self-efficacy.

Healthcare professions, such as nursing and physical therapy, have developed self-efficacy tools to address the self-efficacy considerations for the acute care setting cultural demands as well as skills and knowledge for delivering care to various populations in this setting. Nursing has various self-efficacy tools related to practitioner confidence and abilities to transition from classroom to clinical practice across practice settings. A systematic review conducted by Abusubhiah et al. [10] identified over 37 standardized self-efficacy tools related to entry into a specific practice setting for nursing. More specific to acute care context, Chang et al. [11] developed the Delirium Care Self-Efficacy (DCSE-I) Scale for assessing nurses' self-efficiency for caring for patients in the intensive care unit with risk of delirium. Overall, the researchers found the scale tool had good psychometric properties such as good internal consistency (*r* = 0.94) and good test–retest reliability (*r* = 0.92; [11]). Researchers noted that this tool can be used to help further address the delirium education and care needs that is facilitated by nursing staff bedside.

Within the physical therapy discipline, Greenwood et al. [12] were interested in investigating whether physical therapy students were prepared before their clinical experiences in the acute care and inpatient environments. The Acute Care Confidence Survey (ACCS) was developed based off of current research, acute care practice references, and entry-level skills required by physical therapy students. There were four themes of the survey that were described as judgment, manual skills, mobility skills, and instruction/communication. A Likert scale was used ranging from 10 to 100 with the scale measuring from *very uncertain* to *very certain*. The study concluded that there was strong internal consistency for the full scale (*α* = 0.91), instruct subscale (*α* = 0.75), and judgment subscale (*α* = 0.86), but the mobility and manual subscales both had the same low level of internal consistency (*α* = 0.22). The test–retest reliability of the entire survey was excellent (*intraclass* *correlation* *coefficient* (ICC) = 0.91) and good for judgment (ICC = 0.75), instruct (ICC = 0.86), and mobility (ICC = 0.83) subscales but poor for the manual (ICC = 0.22) subscale. Although the ACCS developed by Greenwood et al. [12] was said to be a reliable and valid tool by their standards, this survey was developed for physical therapy student use only due to the questions being specific to PT skills in acute care. While other scales such the Self-Efficacy for Therapeutic Use of Self (SETUS) Questionnaire [13] and the Self-Efficacy Gauge [14] are OT-specific scales, the former was designed to self-assess practitioners' confidence in their therapeutic use of self without regard to context, and the later is a scale that assesses OT clients' perceived self-efficacy in their current occupational performance skills.

### 1.2. Aim

Although there is a growing body of literature reporting self-efficacy scales among OT practitioners and clients and other healthcare professions working in acute care, there are no reliable tools that evaluate self-efficacy specific to acute care–focused abilities and knowledge within the occupational therapy profession. The researcher's primary aim was to develop and evaluate the internal consistency and test–retest reliability of a new tool, the *Occupational Therapy Acute Care Self-Efficacy Scale* (*OTACSES*). A secondary aim was to determine, via distribution-based methods, the minimal clinically important difference (MCID) of the tool and its subscales related to abilities and knowledge for acute care service delivery that could be used among occupational therapy practitioners and educators for preparation and training related to the acute care setting.

## 2. Materials and Methods

### 2.1. Design

This institutional review board–approved (#1702E07841) measurement development and testing study was conducted in three phases. These included (1) conceptualization and item generation, (2) item reduction, and (3) assessment of test–retest reliability and precision.

### 2.2. Tool Development

#### 2.2.1. Phase I: Conceptualization and Item Generation

Bandura's [15] concept of self-efficacy served as the foundation for the development of OTACSES. A review of the literature identified key components of acute care occupational therapy. Individual qualitative face-to-face interviews were conducted with a sample of six acute care occupational therapists (mean years of practice = 10.5, SD = 1.2) to identify the essentials of acute care OT practice. A sample of six participants is reported to be adequate for qualitative studies [16]. Interview data were analyzed through qualitative content analysis. Through these interviews, two components of self-efficacy were identified: (i) confidence that acute care abilities are adequate and (ii) confidence that required background and procedural knowledge are adequate. See Table 1 for supporting quotes. A total of 11 items were generated describing important knowledge and abilities for OT acute care with seven specific to “abilities” (i.e., “abilities” subscale) and four specific to “knowledge” (i.e., “knowledge” subscale). Responses to each item were collected via a 7-point Likert scale. A 7-point Likert rating scale (i.e., 1 = *very high confidence* to 7 = *very low confidence*) was chosen due to its superior reliability to that of 5-point scales [17], its superior accuracy, and its appropriateness for use in electronic surveys [18].

#### 2.2.2. Phase II: Item Reduction

Phases II and III involved the administration of OTACSES to occupational therapy students from an Accreditation Council for Occupational Therapy Education (ACOTE)–accredited master's program who were 18 or older and could read, write, and understand English. Following the informed consent process, the OTACSES was administered via an online survey database, the REDCap survey management system. Following the administration and collection of responses to the original 11 items, a classical test theory (CTT) psychometric analysis was conducted, where corrected item-total correlation coefficients below 0.4 were considered insufficient and the particular item would be removed to improve internal consistency [19]. This approach has been described to be comparable to that of Rasch analysis [20]. After which, the internal consistency of each subscale was assessed. Internal consistency is the degree to which items of the OTACSES measure the various aspects of the same characteristic and nothing else [21]. Cronbach's alpha coefficient was used to assess internal consistency. Cronbach's alpha coefficient was chosen because it is the most commonly used statistic for measuring internal consistency [21]. Generally, a score between 0.70 and 0.95 indicates a strong internal consistency, while a score > 0.95 may indicate redundancy in test questions [22]. When Cronbach's alpha exceeded 0.95, item reduction would be employed to eliminate any redundancy of items. Data were analyzed using IBM SPSS for Windows (Version 27.0., IBM Corp., Armonk, NY).

#### 2.2.3. Phase III: Psychometric Evaluation: Test–Retest Reliability, Precision, and MCID

During Phase III, the Consensus-based Standards for the Selection of Health Measurement Instruments (COSMIN) guidelines [23] were followed when designing and reporting our methods and findings. An a priori sample size calculation revealed that 33 participants were needed for sufficient statistical power where the lowest acceptable ICC was 0.75 and an anticipated ICC was 0.90 (beta = 0.20; alpha = 0.05) [24].

The OTACSES was administered to students twice, initially at the beginning and then again at the end of the first week of the 15-week entry-level course. To create a stable testing condition and prevent recall bias, no instruction relevant to the topic was delivered during the testing period, the retest occurred within 1 week, and the students were blinded to their initial responses. The test–retest reliability of the subscale and total scores was tested via the ICC (ICC_3,1_) [25]. Higher ICC values indicate stronger reliability, where an ICC of 1 signifies perfect agreement and 0 signifies solely random agreement. Koo and Li [25] suggest that ICCs below 0.40 imply *poor* reliability, those between 0.5 and 0.74 suggest *moderate* reliability, those between 0.75 and 0.89 indicate *good* reliability, and those 0.90 or above suggest *excellent* reliability (Table 1).

Precision was assessed using standard error of measurement (SEM_90_). The SEM is the product of the pooled standard deviation of the trials and (1-ICC). The SEM values were normalized as percentages of the range of scores (i.e., SEM%). Authors have suggested that SEM% values less than 15% indicate acceptable precision [26]. Additionally, the minimal detectable change (MDC), also referred to as the smallest real difference (SRD), a distribution-based method for establishing the MCID, was determined to ascertain the threshold for meaningful change of the measure [27]. The formula for calculating MDC is MDC = (1.96∗√2)∗SEM. Data were analyzed using IBM SPSS for Windows (Version 27.0., IBM Corp., Armonk, NY), with the significance level set to *p* < 0.05. Finally, descriptive statistics were used to characterize the sample and survey results.

## 3. Results

### 3.1. Participants

Forty-eight second semester master's of occupational therapy students were invited to participate; 47 agreed to participate, and all 47 completed each phase of the study. There were 42 females and 5 males, with a mean age of 24 (range 22–27) years and a mean GPA of 3.66. Forty-six students identified as white and one as biracial. At the time of participation, no students had engaged in acute care-specific education, training, or fieldwork within the curriculum. Forty-five percent of the students had no acute care experience, 43% had less than 6 months, 8% had 6 months to 3 years of experience, and 4% had 5 years or more experience. Experiences ranged from observation and volunteer work to inpatient OT assistant or phlebotomy work.

### 3.2. Internal Consistency

The lowest item-total correlations were *r* = 0.83 and *r* = 0.87 for the abilities (*n* = 7) and knowledge (*n* = 4) subscales, respectively, and, as such, no items were initially removed from the scale. When assessing internal consistency of each subscale, the abilities subscale (*α* = 0.96) and knowledge subscale (*α* = 0.92) demonstrated very high internal consistency. However, because Cronbach's alpha of the abilities subscale exceeded 0.95, there appeared to be some redundancy among the items. After removing the item “Interviewing a client in a hospital setting,” an item with a high interitem association (*r* = 0.91) with “Gathering necessary information to create an occupational profile of your client in a hospital setting,” Cronbach's alpha fell into the acceptable range (*α* = 0.94). Conceptually speaking, given that the occupational profile is gathered through interview, this item reduction was deemed appropriate by the developers. The finalized internal consistency results for each subscale are presented in Table 2, and the final version of the OTACSES is located in Figure A1 (see Appendix A).

### 3.3. Test–Retest Reliability, Precision, and MCID

The average time between self-assessments was 4.6 (SD 1.7) days. Descriptive, reliability, and precision results for the subscale and total scores of the OTACSES are reported in Table 2. The ICC scores for the abilities, knowledge, and total scores were 0.93, 0.90, and 0.92, respectively, indicating excellent test–retest reliability for each. The SEM for the abilities, knowledge, and total scores was 2.57, 1.42, and 3.38, respectively. When looking at the SEM as a percentage of the range of scores (i.e., SEM%), the values ranged from 8.7% to 10.7%, indicating that the precision of subscale and total scores was acceptable [26]. The MDC values were 5.03, 2.79, and 6.63 for the abilities, knowledge, and total scores, respectively.

## 4. Discussion

A large percentage of OT practitioners work in nuanced, hospital-based settings. Self-efficacy is known to influence the practice of healthcare students and professionals alike. However, at the time of this study, there was no OT self-efficacy scale specific to acute care contexts. In this study, we sought to develop and assess the reliability and precision of an OT-specific acute care self-efficacy scale for entry-level OT students.

The finalized 10-item version of the OTACSES contains two subscales that assess knowledge and abilities domains, each demonstrating excellent internal consistency. In addition to the subscale scores, a total score is provided, and the test–retest reliability of each subscale and the total score is excellent as per the criteria described by Koo and Li [25]. Thus, the evaluator (e.g., OT educator, OT clinical instructor, and OT researcher) can be certain that the OTACSES has subscales that measure two distinct constructs and is a tool that yields stable scores across time [21]. In addition, our results help to describe the measurement error inherent to the tool. As such, for an evaluator to be confident that change in the scale over time is not due to error but is instead due to, for example, an OT simulation or fieldwork, the change in the scores must at least surpass the SEM. Further, for the evaluator to be assured that the change is of practical meaning to the student, it must exceed the MDC. Finally, as demonstrated by the SEM% analyses, the subscale and total scores all have measurement error that falls into an acceptable range in that the error accounts for less than 15% of the range of scores captured by the scale.

Our findings are fairly consistent with those of other investigations of acute care self-efficacy scales. Chang et al. [11] developed a self-efficacy scale to assess novice and experienced nurses' self-efficacy when working in the intensive care unit. While the scale has strong psychometrics, the survey is only specific to the topic of delirium; many of the items within address topics that are not within our scope of practice, and its measurement properties are only specific to its Chinese version [28]. Greenwood et al. [12] developed a physical therapy–specific acute care self-efficacy scale with excellent internal validity; however, the test–retest reliability of their total and subscale scores ranged from excellent to poor. Although the scale has some strong psychometrics, its subscales have variable reliability, do not include important aspects of the OT process, and seemingly include items that are not often within our scope of practice (e.g., assessing knee flexion range of motion). For these reasons and given its notable measurement properties, we offer the OTACSES as an alternative self-efficacy scale for acute care OT students.

### 4.1. Study Limitations

This study has some limitations that should be considered. First, although the sample size was sufficient to analyze results, a larger sample may have enhanced the robustness of our findings. Second, the survey was built based upon the acute care practices of occupational therapy practitioners working in the United States and may not be appropriate to use in other countries. Third, the sample was comprised of master's of OT students, so although the scale would seem to be appropriate for other relevant groups (e.g., entry-level occupational therapists), further psychometric testing is needed before it can be used in these groups. Finally, our approach to establishing thresholds for meaningful change (i.e., MDC) used a distribution-based approach rather than an anchor-based approach, and some argue that the distribution-based approach does not provide a robust enough indication of the importance of the observed change [29].

### 4.2. Future Research

Further research on the validity and reliability of the OTACSES among practitioners working in acute care is recommended; this might include, for example, examining the construct validity of this scale against other similar scales. Future research should also explore the responsiveness of OTACSES to OT student and practitioner training (e.g., exploring of self-efficacy among occupational therapy students participating in acute care fieldwork experiences or simulation or the effects of a fellowship program for OT practitioners). Furthermore, future research should also explore the scale's threshold for meaningful change via the use of anchor-based methods.

### 4.3. Implications for Occupational Therapy Practice

The findings of this study have the following implications for occupational therapy practice:

• To the authors knowledge, the OTACSES is the first assessment designed to measure OT students' perceived knowledge and abilities relevant to acute care practice.
• OTACSES has excellent reliability, acceptable precision, and established parameters for meaningful change.
• Occupational therapy educators may use the tool for summative and/or formative purposes when evaluating student growth during acute care didactic and/or experiential learning.

## 5. Conclusion

The newly developed OTACSES is a reliable and precise educational tool for OT educators and researchers to measure change in OT student self-efficacy in abilities and knowledge specific to the acute care context. The OTACSES may be used in relevant classroom and/or experiential learning contexts to identify areas of low student self-efficacy and to assess their response to training. Further psychometric testing is recommended to validate the tool's use among entry-level acute care occupational therapy practitioners.

## Figures and Tables

**Figure 1:**
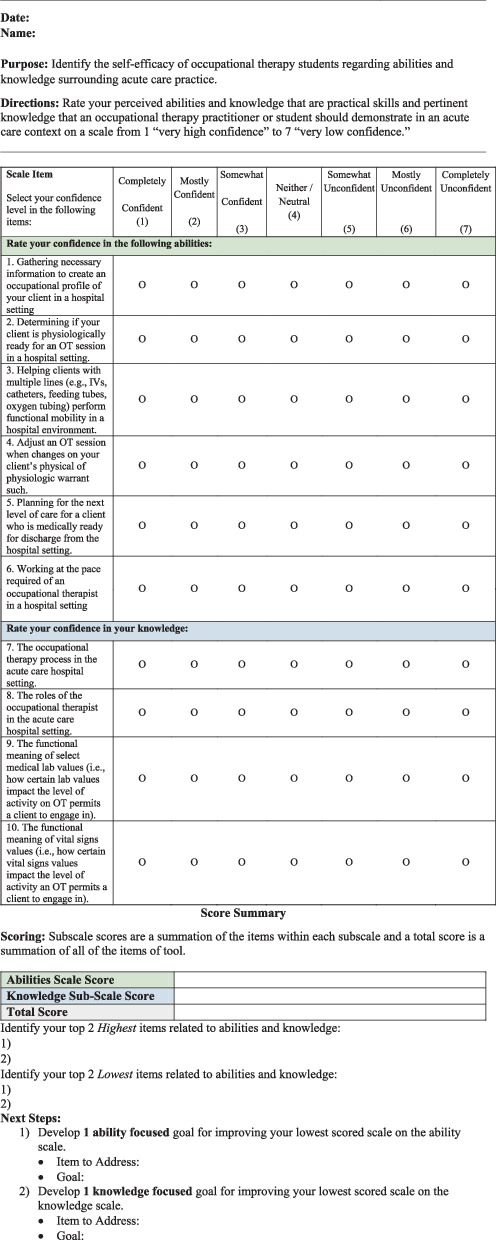
Occupational Therapy Acute Care Self-Efficacy Scale (OTACSES) developed by Corey McGee, PhD, CHT, OTR/L and Hannah Oldenburg, EdD, OTR/L, BCPR.

**Table 1:** Main themes and supporting quotes.

**Subthemes**	**Quotes**
Theme 1: Abilities	
1.1. Gathering necessary information to create an occupational profile of your client in a hospital setting	“chart reviews are important to gather necessary social and medical information, especially when patients are not alert or good historians,” “OTs must learn about the home environment and support systems when planning for discharge,” “gathering information on [premorbid] activity levels must be performed to assess change in disability levels”
1.2. Interviewing a client in a hospital setting	“conducting a thorough interview is needed for discharge planning,” “I will conduct a chart review to learn of any medical precautions,” “chart reviews are an essential part of my process”
1.3. Determining if your client is physiologically ready for an OT session in a hospital setting	“I assess oximetry and BP to anticipate activity tolerance and monitor treatment response,” “I will actively monitor heart rate on my sedated burn patients when ranging them to monitor for pain,” “when a patient has yet to mobilize, I will perform orthostatics and monitor symptoms to assess for hypotension”
1.4. Helping clients with multiple lines (IVs, catheters, feeding tubes, etc.) perform functional mobility in a hospital environment	“I've seen new grads accidently pull out catheters and art(erial) lines when mobilizing patients,”“H.O.B. [head of bed] restrictions must be maintained when performing bed mobility when an N.G. [nasogastric] tube is in place,” “most of my patients have at least 3 or 4 lines [in place] when I work with them”
1.5. Adjust an OT session when changes on your client's physical of physiologic warrant such	“my [intervention] plans are at the mercy of recent changes in medical status or how their [patients'] vitals change [during the session],” “I will sometimes use imagery or deep breathing when a patient's pain increases during a session but when significantly increasing, I will inform nursing and ask if pain medication might be administered,” “depending on the patient's diagnosis or how recently they have undergone a procedure, activity tolerance may fluctuate so I may need to grade down the intensity of things or hold treatment altogether”
1.6. Planning for the next level of care for a client who is medically ready for discharge from the hospital setting	“we [OT] have a different understanding of our patients' and [therefore] we often have different thoughts on discharge [disposition],” “discharge planning is a primary responsibility of OT and begins immediately [upon admission],” “OTs bring an essential lens to the care team when planning for discharge”
1.7. Working at the pace required of an occupational therapist in a hospital setting	“working in acute care can be a grind,” “I spend at least 80% of my time on my feet,” “it took me 6 months or more to build my stamina”
Theme 2: Knowledge	
2.1. The occupational therapy process in the acute care hospital setting	“it took me a while to learn the flow of things,” “I have to do bottom-up evaluations when patients are unable to mobilize” “eval[uation] and treatment looks very different in acute care”
2.2. The roles of the occupational therapist in the acute care hospital setting	“I might be the first to evaluate mobility if PT has yet to see the patient,” “our scope is often limited to evaluating basic ADLs and functional mobility when planning for discharge,” “our clients are often medically ready for discharge so we may only see them 1x to recommend a discharge disposition”
2.3. The functional meaning of select medical lab values (i.e., how certain lab values impact the level of activity on OT permits a client to engage in)	“interpreting lab values such as INR [international normalized ratio], PTT [partial thromboplastin time], and hemoglobin is essential for safety,” “I will often consult the nurse prior to a session if a diabetic patient's blood glucose is below 100 [mg/dl] because their glucose might drop [during activity],” “patients with low hemoglobin, hematocrit, and sodium are at risk for falls due to hypotension”
2.4. The functional meaning of vital signs values (i.e., how certain vital signs values impact the level of activity an OT permits a client to engage in)	“in the neuro[logical] ICU [intensive care unit], ICP [intracranial pressure] must generally be kept between 5 and 15 mmHg during sessions,” “changes in SBP [systolic blood pressure] and DBP [diastolic blood pressure] of 20 and 10 mmHg [respectively] indicate [orthostatic] hypotension,” “sudden changes in heart rate might indicate pain or anxiety,” “it's important to understand the effects of current drug therapy on a patient's vital signs to plan treatment”

**Table 2:** Descriptive, internal consistency, reliability, and precision results for the Occupational Therapy Acute Care Self-Efficacy Scale (OTACSES) for Time 1 and Time 2 (*N* = 47).

**Abilities subscale**	**Test**	**Retest**	**Reliability**	**Precision**		**Internal consistency**
**Please rate your confidence in your following abilities:**	**Time 1** **M** ** (SD)**	**Time 2** **M** ** (SD)**	**ICC (95% CI)**	**SEM (SEM%)**	**MDC**	**Cronbach's alpha**

Gathering necessary information to create an occupational profile of your client in a hospital setting	4.1 (1.4)	4.2 (1.4)				
Determining if your client is physiologically ready for an OT session in a hospital setting	4.6 (1.5)	4.6 (1.7)				
Helping clients with multiple lines (IVs, catheters, feeding tubes, etc.) perform functional mobility in a hospital environment	4.4 (1.7)	4.4 (1.7)				
Adjust an OT session when changes on your client's physical of physiologic warrant such	4.3 (1.4)	4.5 (1.6)				
Planning for the next level of care for a client who is medically ready for discharge from the hospital setting	4.7 (1.6)	4.8 (1.6)				
Working at the pace required of an occupational therapist in a hospital setting	4.4 (1.7)	4.3 (1.6)				
Total abilities subscale score	30.8 (9.7)	31.1 (10.2)	0.93 (0.87–0.96)	2.57 (10.7)	5.03	0.94

**Knowledge subscale**	**Test**	**Retest**	**Reliability**	**Precision**		**Internal consistency**
**Please rate your knowledge on the following items:**	**Time 1** **M** ** (SD)**	**Time 2** **M** ** (SD)**	**ICC (95% CI)**	**SEM (SEM%)**	**MDC**	**Cronbach's alpha**

The occupational therapy process in the acute care hospital setting	3.9 (1.3)	4.0 (1.4)				
The roles of the occupational therapist in the acute care hospital setting	3.7 (1.1)	3.9 (1.4)				
The functional meaning of select medical lab values (i.e., how certain lab values impact the level of activity on OT permits a client to engage in)	4.3 (1.2)	4.3 (1.4)				
The functional meaning of vital signs values (i.e., how certain vital signs values impact the level of activity an OT permits a client to engage in)	4.1 (1.3)	4.4 (1.5)				
Total knowledge subscale score	15.9 (4.5)	16.6 (5.3)	0.90 (0.82–0.95)	1.42 (9.5)	2.79	0.92
Total score	46.8 (13.8)	47.7 (15.1)	0.94 (0.88–0.97)	3.38 (8.7)	6.63	

## Data Availability

The data that support the findings of this study are available from the corresponding author upon reasonable request.
